# Endoscopic Management of Complex Duodenal Ulcer Bleeding Using Over‐the‐Scope Clips: Clinical Insights From a Regional Core Hospital

**DOI:** 10.1002/deo2.70153

**Published:** 2025-06-05

**Authors:** Akihiro Maruyama, Hirotaka Takeshima, Hiroshi Nakayabu, Hiroki Kato, Shintaro Tominaga, Makoto Kobayashi

**Affiliations:** ^1^ Department of Gastroenterology Yokkaichi Municipal Hospital Mie Japan

**Keywords:** duodenal ulcer, Forrest Ia ulcers, over‐the‐scope clips, posterior wall lesions, upper gastrointestinal bleeding

## Abstract

**Objectives:**

Over‐the‐scope clips (OTSCs) are considered an effective endoscopic tool for managing upper gastrointestinal bleeding, including duodenal ulcers, mostly based on data from high‐volume centers with expert endoscopists. This study aimed to evaluate the clinical safety of OTSCs in regional hospital backgrounds and identify the factors associated with unsuccessful hemostasis.

**Methods:**

We conducted a retrospective study of 30 patients with duodenal ulcer bleeding who underwent OTSC placement at a regional core hospital in Japan between April 2014 and January 2025. Clinical outcomes, rebleeding rates, complications, and subgroup analyses by ulcer location, Forrest classification, and operator experience were evaluated.

**Results:**

Primary hemostasis was achieved in 28 of 30 patients (93.3%). Rebleeding occurred in two cases (6.7%) but was successfully managed endoscopically. Both hemostasis failures involved Forrest Ia ulcers on the posterior duodenal wall. Subgroup analysis revealed significantly lower success rates for Forrest Ia (66.7%) and posterior wall lesions (33.3%). No significant differences in outcomes were observed between experienced and less‐experienced endoscopists. Postprocedural complications included mild pancreatitis and duodenal stricture, both managed conservatively. OTSC was used as a first‐line modality in 10 cases and as salvage therapy in 20, with all failures occurring in the latter.

**Conclusion:**

OTSC is a safe and effective hemostatic modality for duodenal ulcer bleeding, even in regional hospitals with limited resources and staffing. It is particularly useful when rapid intervention is required and alternative treatments are not readily available. However, anatomical challenges such as posterior wall location and Forrest Ia classification may predict technical failure.

## 1 Introduction

Endoscopic hemostasis remains the standard for the management of upper gastrointestinal bleeding, with peptic ulcer disease accounting for a substantial proportion of cases [1, 2]. Duodenal ulcer bleeding poses particular challenges owing to its anatomical complexity, rich vascular supply, and proximity to critical structures, such as the ampulla of Vater. Conventional modalities, including through‐the‐scope clips (TTSC), thermal coagulation, and injection therapy, have improved overall clinical outcomes [3]. However, achieving durable hemostasis in duodenal ulcer treatment remains difficult in certain cases.

The Over‐the‐Scope Clip (OTSC; Ovesco Endoscopy, Tübingen, Germany) represents a major innovation in endoscopic hemostasis. By enabling full‐thickness tissue capture and deep vessel compression, OTSC provides distinct advantages over traditional TTSC systems, particularly for refractory bleeding or challenging lesions [4]. Recent guidelines from the European Society of Gastrointestinal Endoscopy (ESGE) endorse OTSC not only as a salvage therapy but also as a primary modality for high‐risk upper gastrointestinal bleeding. These endorsements reflect the growing recognition of OTSC's potential to significantly impact the management of gastrointestinal bleeding.

While several studies have reported favorable hemostasis rates using OTSCs for duodenal ulcers [5, 6, 7], most existing evidence has been derived from high‐volume centers with expert endoscopists and advanced resources. Consequently, the generalizability of these findings to real‐world clinical environments, especially in resource‐limited institutions, is not feasible.

Regional core hospitals play an essential role in providing emergency care to geographically dispersed populations. These hospitals often face unique challenges, including a higher proportion of elderly patients with multiple comorbidities, reduced staffing during emergencies, and greater reliance on single‐operator endoscopy. In such settings, the feasibility, technical challenges, and safety profile of OTSC therapy warrant dedicated evaluation, particularly when procedures are performed under constraints not typically encountered in tertiary care centers.

This study aimed to assess the clinical safety of OTSC use for duodenal ulcer bleeding in a real‐world, resource‐constrained environment. By analyzing outcomes based on ulcer location, Forrest classification, and procedural factors, we sought to provide practical insights into the applicability of OTSCs beyond specialized centers, highlighting their potential to enhance emergency care in diverse healthcare settings.

## 2 Materials and Methods

### 2.1 Study Design and Patient Selection

This retrospective study reviewed 30 consecutive cases of duodenal ulcer bleeding treated with OTSCs between April 2014 and January 2025 at our institution. A total of 311 patients underwent endoscopic hemostasis for duodenal ulcer bleeding during the study period. Among these, 30 patients (9.6%) received OTSC therapy. The remaining patients underwent hemostasis using conventional modalities such as thermal coagulation, epinephrine injection, or TTSC and were excluded from the present study. The decision to use OTSC was made at the discretion of the endoscopist based on clinical judgment, taking into account factors such as bleeding severity, ulcer location, vessel size, and the expected efficacy of standard techniques. The inclusion criteria were endoscopic confirmation of duodenal ulcer bleeding and the application of OTSCs for hemostasis. Patients with malignant ulcers, non‐bleeding ulcers (Forrest III), or bleeding from non‐duodenal sources were excluded. Ten patients (33.3%) received OTSC as first‐line hemostatic therapy, while twenty patients (66.7%) underwent OTSC placement as salvage therapy following failure of conventional methods. The data collected included patient demographics, lesion characteristics, Forrest classification, nonsteroidal anti‐inflammatory drug (NSAID) or steroid use, dialysis status, operator experience, and procedural details. This study was approved by the ethics committee of Yokkaichi Municipal Hospital (Mie, Japan), and all patients provided informed consent.

### 2.2 OTSC Procedure

**FIGURE 1:**
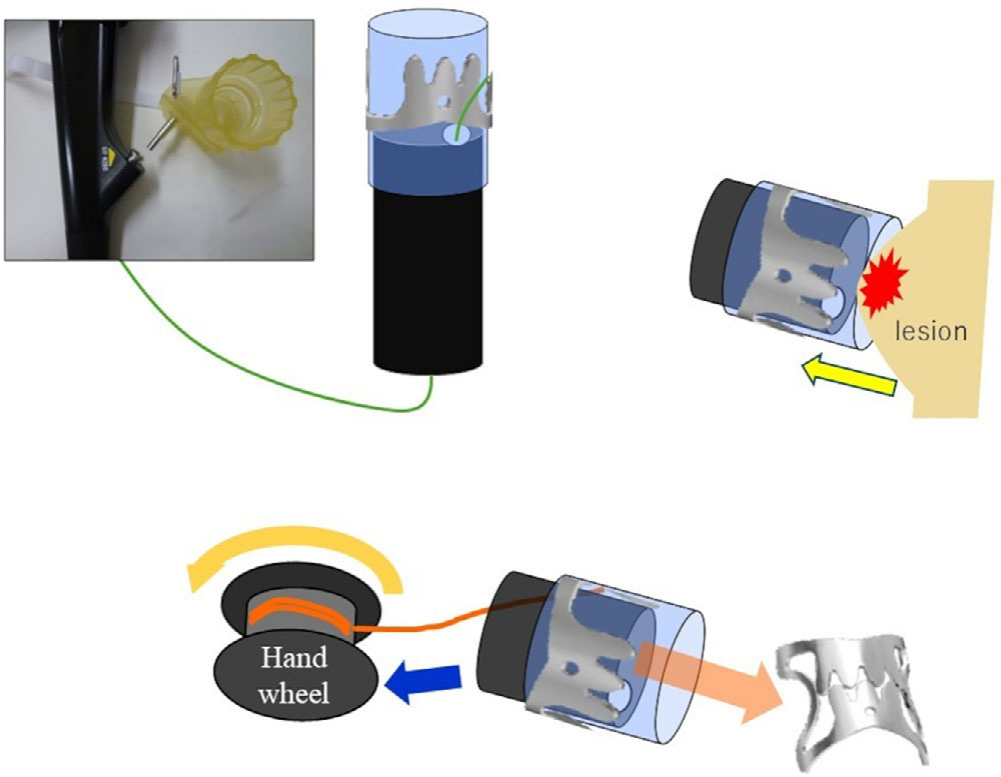
Schematic representation of the over‐the‐scope clip deployment procedure. A large over‐the‐scope clip (OTSC) was mounted at the distal end of the applicator cap attached to the tip of the endoscope. A tensioning thread connected to the OTSC ran through the working channel of the endoscope and was linked to the hand wheel positioned at the accessory port. Suction was applied to draw the target tissue into the cap after positioning the applicator cap over the bleeding lesions. The hand wheel was then rotated to tighten the thread, release the clip, and achieve full‐thickness tissue closure for hemostasis.

OTSC devices were deployed using standard endoscopic techniques. Following lesion identification, suction was applied to capture the tissue surrounding the applicator cap (Figure 1). Auxiliary tools, such as the Twin Grasper (Ovesco Endoscopy, Tübingen, Germany), were used in two cases (6.7%), while OTSCs were deployed without accessory devices in the remaining 28 cases (93.3%). All procedures utilized 9‐mm, traumatic‐type (t‐type) OTSCs. Procedure time was defined as the interval from initial endoscope insertion to successful OTSC deployment.

### 2.3 Patient Characteristics

Thirty patients with a mean age of 74.3 years (range: 55–92 years) were included, comprising 18 males and 12 females (Table 1). Nine patients (30.0%) were receiving NSAIDs, three (10.0%) were receiving systemic corticosteroids, and five (16.7%) were undergoing maintenance dialysis. The most frequent site of bleeding was the descending section of the duodenum, which was observed in 11 patients (36.7%). Bleeding from the duodenal bulb was further classified by wall involvement as follows: anterior wall, eight cases (26.7%); posterior wall, three cases (10.0%); inferior wall, two cases (6.7%); and superior wall, one case (3.3%). Additional bleeding sites included the superior duodenal angle (three patients, 10.0%), horizontal section (one patient, 3.3%), and ascending section (one patient, 3.3%). According to the Forrest classification, six patients (20.0%) presented with type Ia bleeding, 20 (66.7%) with type Ib, and four (13.3%) with type IIa bleeding; no cases of type IIb or higher bleeding were observed.

**TABLE 1:** Patient characteristics.

Variable	Value
Patients, *n*	30
Mean age, years (range)	74.3 (55–92)
Male sex, *n* (%)	18 (60.0%)
NSAID use, *n* (%)	9 (30.0%)
Steroid use, *n* (%)	3 (10.0%)
Dialysis, *n* (%)	5 (16.7%)
Antithrombotic use, *n* (%)	7 (23.3%)
Forrest classification—Ia, *n* (%)	6 (20.0%)
Forrest classification—Ib, *n* (%)	20 (66.7%)
Forrest classification—IIa, *n* (%)	4 (13.3%)
Ulcer location—Duodenal bulb, anterior wall, *n* (%)	8 (26.7%)
Ulcer location—Duodenal bulb, posterior wall, *n* (%)	3 (10.0%)
Ulcer location—Duodenal bulb, inferior wall, *n* (%)	2 (6.7%)
Ulcer location—Duodenal bulb, superior wall, *n* (%)	1 (3.3%)
Ulcer location—Superior duodenal angle, *n* (%)	3 (10.0%)
Ulcer location—Descending section, *n* (%)	11 (36.7%)
Ulcer location—Horizontal section, *n* (%)	1 (3.3%)
Ulcer location—Ascending section, *n* (%)	1 (3.3%)

### 2.4 Outcome Measures

The primary outcomes were primary hemostasis and rebleeding rates within 3 months. Primary hemostasis was defined as successful endoscopic hemostasis without evidence of rebleeding within 72 h of OTSC placement. Secondary outcomes included complications (such as pancreatitis and stricture formation) and the total procedure time. Subgroup analyses assessed the impact of patient and procedural factors, including age (≥ 75 vs. < 75 years), NSAID or steroid use, antithrombotic use, dialysis status, Forrest classification, operator experience (≥ 5 vs. < 5 prior OTSC procedures), and duodenal location.

### 2.5 Statistical Analysis

Categorical variables, including primary hemostasis and subgroup comparisons, were analyzed using Fisher's exact test. For continuous variables, such as procedure time, intergroup comparisons were conducted using the Mann–Whitney U test, given the non‐parametric nature and small sample size of the data. Statistical significance was set at *p* < 0.05. All analyses were performed using Python (version 3.12) with the appropriate statistical packages.

## 3 Results

### 3.1 Overall Outcomes

Primary hemostasis was achieved in 28/30 (93.3%) patients who underwent OTSC placement for duodenal bleeding (Table 2). OTSC was used as first‐line therapy in 10 cases (33.3%) and as salvage therapy following failure of conventional modalities in 20 cases (66.7%).

**TABLE 2:** Summary of clinical outcome.

	Overall (*n* = 30)	First‐line use (*n* = 10)	Salvage use (*n* = 20)
Initial hemostasis achieved	28 (93.3%)	10 (100%)	18 (90.0%)
Rebleeding	2 (6.7%)	0 (0%)	2 (10.0%)
Adverse events	2 (6.7%)	1 (10.0%)^†^	1 (5.0%)^‡^
Procedure time, median (IQR), min	33.0 (22.8–40.8)	31.0 (21.8–39.3)	32.5 (24.3–48.0)

^†^ Acute pancreatitis.

^‡^ Duodenal stenosis.

**FIGURE 2:**
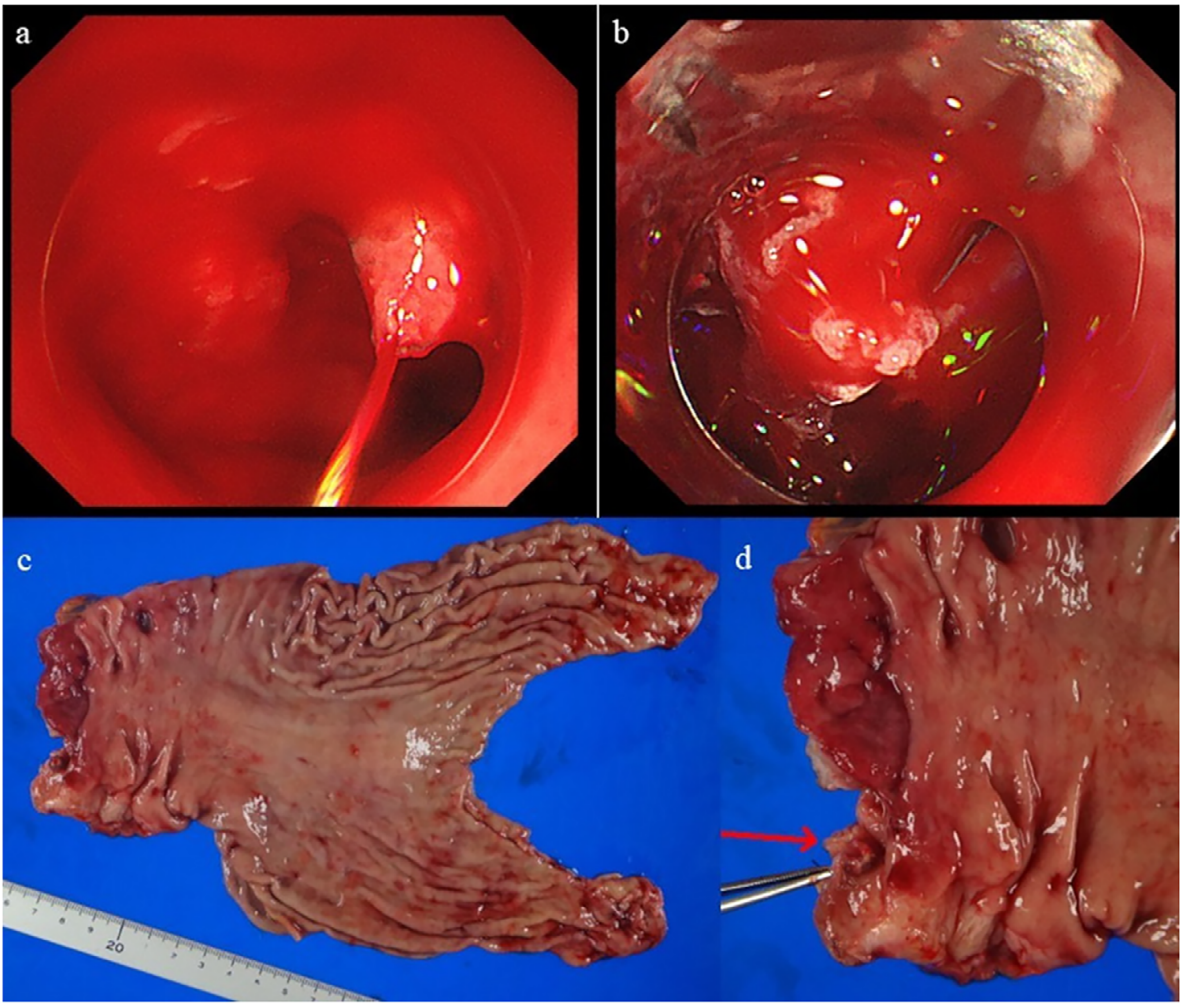
Representative case of over‐the‐scope clip (OTSC) failure in a posterior duodenal bulb ulcer. (a) Endoscopic view showing a spurting hemorrhagic ulcer located on the posterior wall of the duodenal bulb. b) Attempted OTSC placement failed to achieve hemostasis due to insufficient suction of surrounding tissue into the applicator cap. (c) The patient subsequently underwent subtotal gastrectomy. (d) Intraoperative ligation of the gastroduodenal artery (GDA) successfully controlled the bleeding originating from an exposed vessel (red arrow).

**FIGURE 3:**
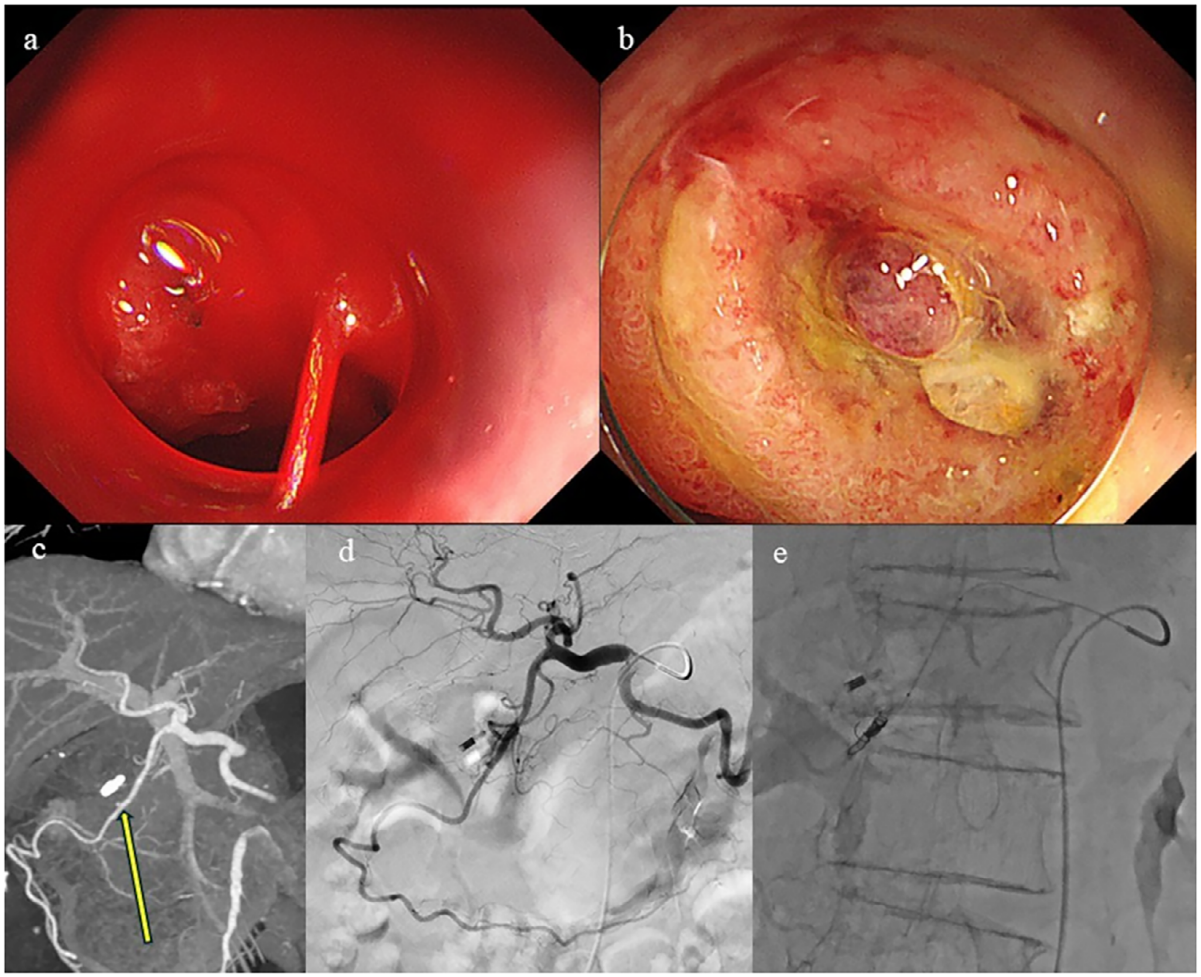
Representative case of failed over‐the‐scope clip (OTSC) placement followed by successful transcatheter arterial embolization (TAE). (a) Endoscopic image showing a spurting hemorrhagic ulcer on the posterior wall of the duodenal bulb. Attempted OTSC placement was unsuccessful due to inadequate suction of surrounding tissue. (b) Temporary hemostasis was achieved using thermal coagulation and topical application of Purastat; however, a pulsatile vessel remained, and only a clip was placed in proximity. Rebleeding occurred two days later, necessitating interventional radiology. (c, d) A pseudoaneurysm was suspected at the origin of the anterior superior pancreaticoduodenal artery, noted as a focal irregular dilatation (yellow arrow) on contrast‐enhanced computed tomography (c) and celiac angiography (d). (e) Coil embolization was performed successfully, achieving definitive hemostasis.

**FIGURE 4:**
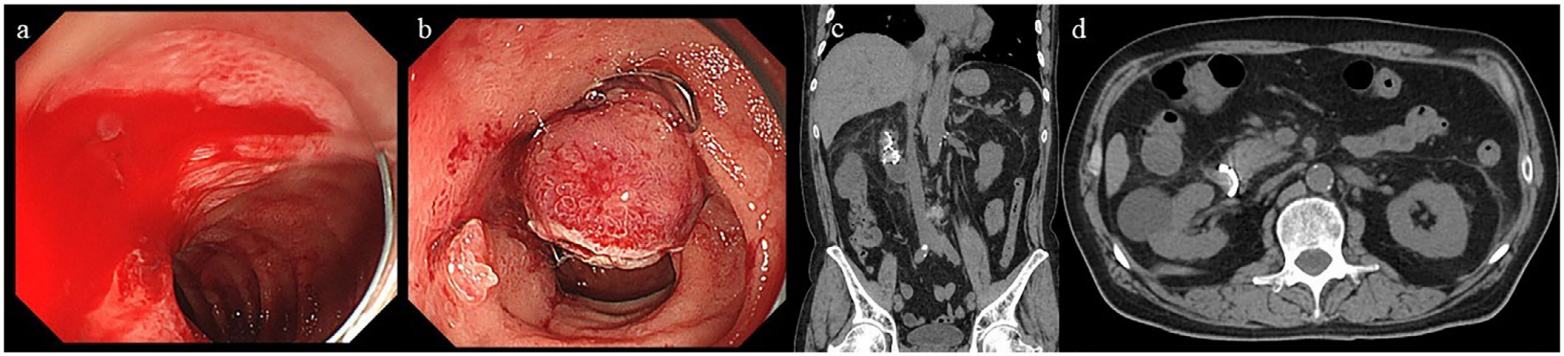
Case of successful hemostasis with over‐the‐scope clip (OTSC) followed by acute pancreatitis. (a) Endoscopic view showing a broad‐based ulcer with oozing hemorrhage in the horizontal section of the duodenum. (b) Two OTSCs were deployed, resulting in successful hemostasis. (c, d) A few days later, the patient developed mild acute pancreatitis. Abdominal computed tomography demonstrated increased fat stranding around the pancreas. (c) Coronal view; (d) axial view.

**FIGURE 5:**
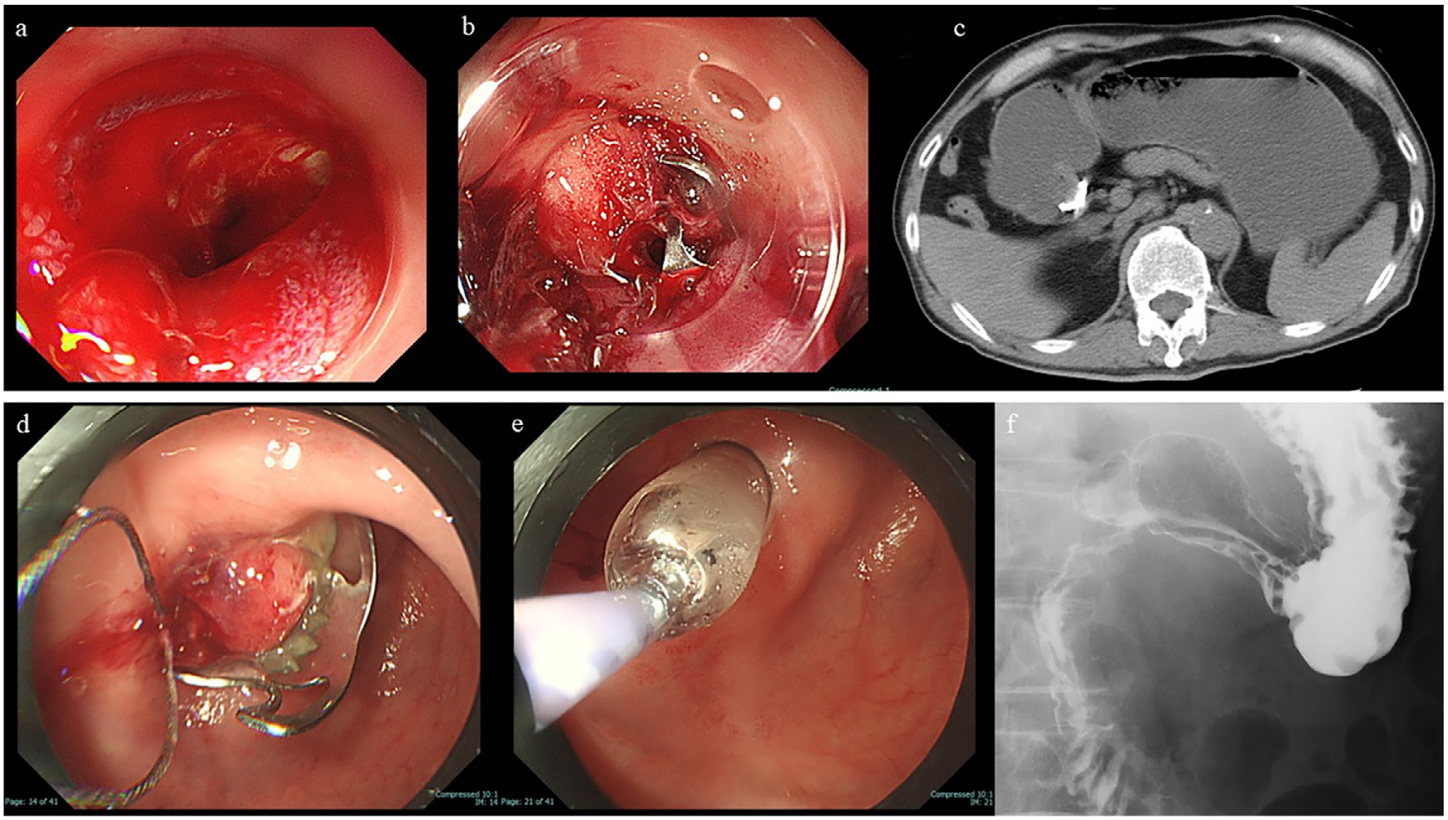
Case of post‐over‐the‐scope clip (post‐OTSC) duodenal stricture managed endoscopically. (a) Endoscopic image showing an oozing ulcer at the superior duodenal angle. (b) Hemostasis was achieved by OTSC placement. (c) On day 48 post‐procedure, upstream bowel dilatation was noted proximal to the OTSC site. (d) The clip was removed using a snare. (e) An endoscopic balloon was performed at the stricture site. (f) Gastrografin fluoroscopy following dilation confirmed satisfactory passage through the duodenum.

Primary hemostasis was achieved in all 10 first‐line cases (100%) and in 18 of 20 salvage cases (90.0%). Additionally, the auxiliary tool, Twin Grasper, was used in two cases (6.7%), of whom, only one achieved hemostasis. Overall, two patients failed to achieve initial hemostasis; one was ultimately subjected to emergency surgery, whereas the other achieved definitive hemostasis through transcatheter arterial embolization (Figures 2 and 3). Rebleeding occurred in two of the 30 initially successful cases (6.7%). The median procedure time for all cases was 33.0 min. Two postprocedural complications were observed: one case was of mild acute pancreatitis, which resolved with conservative management within 1 week; and the other case was of duodenal stricture, in which the OTSC was endoscopically removed, followed by successful endoscopic balloon dilation, leading to symptom resolution without surgical intervention (Figures 4 and 5). No delayed perforations were observed.

### 3.2 Subgroup Analyses

#### 3.2.1 Ulcer Location

Primary hemostasis was significantly less frequent in posterior wall lesions (33.3%, 1/3 cases) than in non‐posterior wall lesions (100%, 27/27 cases; *p* = 0.0069) (Table 3). The median procedure time was 40.0 min for posterior wall lesions and 30.0 min for other lesions (*p* = 0.2836).

**TABLE 3:** Subgroup analysis.

Factor	Subgroup	Hemostasis rate (%)	Median procedure time (min)	*p*‐Value (Hemostasis)	*p*‐Value (Procedure time)
Ulcer location	Others	100.0% (27/27)	30	0.0069	0.2836
Ulcer location	Posterior Wall	33.3% (1/3)	40		
Forrest classification	1a	66.7% (4/6)	39	0.0345	0.1077
Forrest classification	Others	100.0% (24/24)	29		
Operator experience	Expert	84.6% (11/13)	36	0.1793	0.5028
Operator experience	Trainee	100.0% (17/17)	29		
Steroid use	Yes	100.0% (3/3)	29	1	0.6781
Steroid use	No	92.6% (25/27)	33		
NSAID use	Yes	88.9% (8/9)	22	0.5172	0.0602
NSAID use	No	95.2% (20/21)	36		
Age group	≥75 years	86.7% (13/15)	37	0.4828	
Age group	<75 years	100.0% (15/15)	30		
Dialysis	Yes	100.0% (5/5)	33	1	0.8893
Dialysis	No	92.0% (23/25)	30		
Antithrombotic use	Yes	100.0% (7/7)	28	1	0.321
Antithrombotic use	No	91.3% (21/23)	38		

NSAID: nonsteroidal anti‐inflammatory drug.

#### 3.2.2 Forrest Classification

The primary hemostasis rate for Forrest 1a lesions was 66.7% (4/6), which was significantly lower than that for lesions of other classifications (100%, 24/24; *p* = 0.0345). The median procedure time was also longer in Forrest 1a cases (39.0 min) than in other cases (29.0 min), although the difference was not statistically significant (*p* = 0.1077).

#### 3.2.3 Operator Experience

Experienced operators (≥ 5 prior OTSC procedures) had a primary hemostasis rate of 84.6% (11/13), whereas less‐experienced operators achieved 100% (17/17; *p* = 0.1793). The median procedure times were 36.0 and 29.0 min, respectively (*p* = 0.5028).

#### 3.2.4 Steroid Use

All three patients receiving corticosteroids achieved hemostasis (100%) compared to 92.6% (25/27) of non‐users (*p* = 1.0000). The median procedure times were comparable (29.0 vs. 33.0 min; *p* = 0.6781).

#### 3.2.5 NSAID Use

The primary hemostasis rate was 88.9% (8/9) in NSAID users and 95.2% (20/21) in non‐users (*p* = 0.5172). NSAID users had a shorter median procedure time (22.0 vs. 36.0 min; *p* = 0.0602), although the difference was not statistically significant.

#### 3.2.6 Age

In patients aged ≥ 75 years (*n* = 15), the primary hemostasis rate was 86.7% (13/15) compared with 100% (15/15) in those aged <75 years (*p* = 0.4828). The median procedure times were slightly longer in the older group (37.0 vs. 30.0 min).

#### 3.2.7 Dialysis Status

Among the dialysis‐dependent patients (*n* = 5), the primary hemostasis rate was 100%, whereas it was 92.0% (23/25) in non‐dialysis patients (*p* = 1.0000). The median procedure times were 33.0 and 30.0 min, respectively (*p* = 0.8893).

#### 3.2.8 Antithrombotic Use

The primary hemostasis rate was 100% (7/7) in patients receiving antithrombotic therapy and 91.3% (21/23) in those not receiving it (*p* = 1.0000). Median procedure times were 28.0 and 38.0 min, respectively (*p* = 0.3210)

## 4 Discussion

This retrospective study evaluated the utility of OTSCs for managing duodenal ulcer bleeding in a regional core hospital setting. Among the 30 patients analyzed, the overall primary hemostasis rate was 93.3%, and rebleeding occurred in 6.7% of initially successful cases. These findings are comparable to those reported in prior large‐scale studies and support the clinical feasibility of OTSC use in non‐tertiary care institutions, and also consistent with previous findings from Kobara et al. and other randomized controlled trials [8, 9, 10, 11], which reported similar efficacy profiles for OTSC, while highlighting its real‐world utility outside of high‐volume academic centers.

Although previous randomized controlled trials have demonstrated the efficacy of OTSCs in upper gastrointestinal bleeding, most were conducted in high‐volume centers with expert endoscopists. In contrast, our study reflects real‐world conditions in a resource‐limited regional hospital, where staffing constraints and urgent care demands often necessitate rapid decision‐making and intervention by general endoscopists. The high hemostasis rate observed, including among cases performed by less‐experienced operators, suggests that OTSCs can be effectively and safely applied even in not‐so‐well‐equipped environments.

Subgroup analysis revealed that lesions located on the posterior wall of the duodenal bulb and those classified as Forrest Ia were significantly associated with technical failure. Both cases of initial hemostasis failure involved posterior wall, and Forrest Ia ulcers and required subsequent surgical or radiological intervention. These findings emphasize the technical difficulty of deploying OTSCs in anatomically challenging locations, particularly where exposure and suctioning of the bleeding point are limited. In one case, even the use of a Twin Grasper failed to facilitate adequate tissue capture, necessitating emergency surgery. In the other, transcatheter arterial embolization achieved definitive hemostasis. Notably, both ulcers were located on the posterior wall, near the transition between the duodenal bulb and the descending portion, an area known for its rigidity and limited compliance. The inability to adequately suction the bleeding site into the cap strongly suggests that the ulcers may have been fibrotic, which has been reported in previous literature as a factor that complicates OTSC application [8]. Moreover, contrast‐enhanced computed tomography revealed a focal irregular dilatation indicative of a pseudoaneurysm at the origin of the anterior superior pancreaticoduodenal artery. When feasible, pre‐procedural imaging may provide valuable information to guide the appropriateness of OTSC application.

Although Forrest Ia lesions have been previously reported as favorable indications for OTSC placement in large‐scale studies, including those by Soriani and Mangiafico [9, 10], our study encountered two failures among Forrest Ia cases. However, given the limited sample size, these findings should be interpreted with caution and not overgeneralized. However, both failed cases involved ulcers on the posterior wall, suggesting that the combination of Forrest Ia bleeding and challenging anatomical location may synergistically increase the technical difficulty. These cases underscore the limitations of OTSCs and necessitate surgical or radiological interventions in select patients [12, 13, 14]. Importantly, prior studies have typically categorized ulcers by location only as “duodenal bulb” without distinguishing anterior, posterior, or transitional positions. Moreover, further accumulation of case data is needed to clarify whether certain combinations pose a higher risk for failure.

Given the rich vascular anatomy of the duodenum, particularly the posterior wall's proximity to large‐caliber vessels from the pancreaticoduodenal arcade and gastroduodenal artery [15, 16], refractoriness to endoscopic clipping in such locations is not surprising. Moreover, the mechanical limitations of the OTSC system, including reduced maneuverability and limited suction in rigid or angulated segments, may hinder its effectiveness in posterior lesions. In such scenarios, early consideration of alternative modalities such as embolization or surgery should be prioritized.

Among the 28 patients who achieved primary hemostasis, two experienced rebleeding. One case occurred on post‐procedural day 5 due to oozing from the inter‐clip gap, which was successfully managed with adjunctive thermal coagulation. The other case involved delayed rebleeding on day 90, where the ulcer had persisted and the OTSC had dislodged; hemostasis was achieved by additional clipping. Neither case experienced further rebleeding during follow‐up. OTSC is highly effective owing to its capacity for full‐thickness compression and deep vessel tamponade, but its design incorporates a small inter‐prong gap to prevent tissue necrosis [17], which may lead to early or delayed rebleeding. Additionally, OTSC being a full‐thickness closure device, further coagulation can be safely performed, even in the thin‐walled duodenum, without risk of perforation. OTSC may also be supplemented with additional clipping, enabling flexible endoscopic management even in cases of partial hemostasis or delayed bleeding [18].

Regarding postprocedural complications, two adverse events were observed. One patient developed mild acute pancreatitis following OTSC placement near the ampulla of Vater, which resolved with conservative management. A similar case has been previously reported in the literature, in which pancreatitis improved after the endoscopic removal of an OTSC [19]. Another patient experienced duodenal luminal narrowing 48 days post‐procedure, requiring OTSC removal and endoscopic balloon dilation. In cases of suspected luminal obstruction or clip misplacement, early endoscopic evaluation and removal should be considered to prevent delayed complications [20, 21]. In particular, given the duodenum's complex anatomical features—such as the presence of the ampulla of Vater and sharp angulations—clinical decisions should not be limited to achieving hemostasis. For instance, in the duodenal bulb, it is important to confirm post‐OTSC luminal patency. In the horizontal portion, postoperative follow‐up using MRCP or CT may be warranted to assess for delayed dilation of the pancreatic or biliary ducts. These anatomical factors highlight the importance of not only technical success but also thoughtful long‐term planning when employing OTSC in duodenal lesions.

OTSC was used as a first‐line hemostatic tool in 10 cases and as salvage therapy in 20. The hemostasis rate remained high in both groups, with failure occurring only in the salvage group. Furthermore, no significant differences in procedural success or duration were observed between experienced and less‐experienced endoscopists, suggesting that OTSC is a reproducible and user‐friendly modality, not demanding expert hands. Additionally, unlike conventional hemostatic techniques such as through‐the‐scope clips or thermal coagulation, OTSC does not require pinpoint targeting of the bleeding vessel. If the bleeding site can be drawn into the cap, effective hemostasis can often be achieved simply by releasing the clip [17, 22], making it a potentially more straightforward option for urgent cases.

Thus, OTSC may serve as a critical endoscopic option during the unavailability of experienced endoscopists, and cases, when interventional radiology or surgical backup may be limited.

This study has some limitations. As a retrospective, single‐center study with a relatively small sample size, generalizability is limited. Similarly, based on the results of only two failed cases, evaluating Forrest Ia and the posterior wall as risk factors may be an overestimation. Selection bias may have occurred, as the decision to use OTSC was at the operator's discretion, and no standardized treatment algorithm was applied. Moreover, lesion size and detailed ulcer morphology were not evaluated. Another limitation is the lack of a control or comparison group, preventing the assessment of the relative efficacy of OTSC. Future studies considering all these limitations are essential to conclude about the superiority of OTSC. Despite these limitations, our study provides valuable insight into the practical application of OTSCs in routine clinical settings and highlights anatomical and procedural factors associated with failure. Additionally, the insufficient literature, including data from local areas of Japan, demonstrating the use of OTSC for managing duodenal ulcer bleeding in a regional core hospital setting, makes this study design advantageous.

In conclusion, OTSCs are effective and safe for duodenal ulcer bleeding even in regional hospitals with limited staffing and resources. This is one of the few studies focusing on OTSC use in a regional hospital context, suggesting factors responsible for failures, thereby contributing valuable real‐world data to the growing literature on advanced endoscopic hemostasis.

## Conflicts of Interest

The authors declare no conflicts of interest.

## Ethics Statement

**Approval of the research protocol by an Institutional Review Board**: Approved by the Ethics Committee of Yokkaichi Municipal Hospital, Japan (Approval No. 2020–4).

## Consent

Oral informed consent was obtained from all patients prior to participation, in accordance with the approved protocol. The consent process was documented in the patients' medical records.

## Clinical Trial Registration

N/A
